# Short- and long-term outcomes following percutaneous coronary intervention in hepatitis C virus seropositive patients

**DOI:** 10.1186/s43044-020-00079-9

**Published:** 2020-07-25

**Authors:** Ahmed Hussein, Mohamed Abdel Ghany, Hossam Eldin M. Mahmoud

**Affiliations:** 1 Department of Internal Medicine, Faculty of Medicine, Sohag University, Nasser city, Sohag 82524 Egypt; 2 Department of Cardiology, Faculty of Medicine, Assiut University, Assiut city, Assiut 71511 Egypt; 3 Department of Internal Medicine, Faculty of Medicine, South Valley University, Qena city, Qena 83511 Egypt

**Keywords:** HCV seropositivity, PCI, MACEs, Clinical in-stent restenosis

## Abstract

**Background:**

Hepatitis C virus (HCV) infection is progressively recognized as a potential atherogenic condition that is associated with coronary artery disease (CAD). Factors that affect the cardiovascular system as diabetes mellitus and dyslipidemia also may affect the outcomes following PCI. So, HCV infection may have an impact on the outcomes following PCI. We aimed to investigate the impact of HCV seropositivity on the outcomes following percutaneous coronary intervention (PCI).

**Results:**

We conducted a multi-center prospective cohort study on 400 patients candidate for elective PCI using drug-eluting stents; 200 patients were HCV seropositive and did not received antiviral treatment, and 200 patients were HCV seronegative. The patients were followed up for 1 year for the development of major adverse cardiovascular events (MACEs) and clinical in-stent restenosis. Multivariate Cox hazard regression analyses for MACEs and clinical in-stent restenosis at 12 months after adjustment for confounding factors showed that HCV seropositivity did not present a higher hazard upon MACEs (adjusted hazard ratio (HR) 0.74; 95% CI 0.41–1.32; *p* value 0.302), the individual cardiovascular outcomes (target lesion revascularization (TLR), target vessel revascularization (TVR), myocardial infarction (MI), cerebrovascular stroke (CVS), stent thrombosis, major bleeding, coronary artery bypass graft (CABG), cardiac death, and non-cardiac death), or the incidence of clinical in-stent restenosis (adjusted HR was 1.70; 95% CI 0.64–4.51; *p* value 0.28) compared to seronegative patients.

**Conclusion:**

HCV seropositivity had no impact on MACEs, individual cardiovascular outcomes, or clinical in-stent restenosis following PCI for a 1 year follow-up period.

## Background

Many theories suggested infection as a tributary risk factor of CAD [1, 2]; the association between infections and CAD has been explained by different direct and indirect mechanisms [2–4]. The acquired information shows the recognizable proof of viral genomes in the atherosclerotic plaques and furthermore proatherogenic impacts of viral disease in cells applicable to atherogenesis (smooth muscle cells, monocyte macrophages, T cells, and endothelial cells) [5]. HCV infection is a major public health burden in Egypt; the Demographic Health Survey (DHS) of 2015 showed that among those aged 15–59 years the national seroprevalence was 10%, which is the highest prevalence rate in the world [6]. It was estimated that the global prevalence of HCV infection is 2–3% [7]. HCV infection is progressively recognized as a potential atherogenic condition. Actually, the chronic HCV disease causes hepatic and systemic inflammation by means of expanded degrees of proatherogenic chemokines and cytokines [8]. Hepatic steatosis is more common in patients with HCV infection, which has many clinical features as those in patients with metabolic syndrome [9, 10]. Hepatic steatosis is associated with endothelial dysfunction and elevated levels of inflammatory markers [11]. Also, HCV infection was suggested to be associated with atherosclerosis [3, 12–14], CAD [15–17], carotid artery disease [18], and stroke [19, 20]. In spite of that, it is still controversial whether there is an association between HCV infection and CAD or not [21–25]. Different studies have shown conflicting results, some studies showed that there is an association between HCV seropositivity and CAD and carotid atherosclerosis [3, 16, 26, 27], other studies showed that there is no association between HCV infection and CAD, carotid atherosclerosis, or risk of myocardial infarction [2, 28–31], while Pothineni et al. reported that HCV infected patients had less obstructive CAD on coronary angiography [32].

We know that the factors have effects on the cardiovascular system as diabetes mellitus and dyslipidemia also affect the outcomes following PCI. It is postulated that infections adversely affect the cardiovascular system. So, HCV infection may have an impact on the outcomes following PCI. Only a limited research has been carried out on this particular topic. Therefore, we conducted this study to investigate the effect of HCV seropositivity on the short- and long-term outcomes following PCI.

## Methods

### Aim of the study

This study was conducted to detect the effect of HCV seropositivity on the short- and long-term outcomes in patients after PCI.

### Design of the study

This was a multi-center prospective cohort study conducted on 400 patients candidate for elective PCI using drug-eluting stents, at three different University Hospitals, Cath. Lab. Unit from May 2016 to November 2018. The participants were classified into two equal groups: group 1 was seropositive for HCV and did not received antiviral treatment, and group 2 was seronegative for HCV.

All subjects provided a written informed consent to participate in the study. The study protocol was approved by the ethics committee at Universities where the study was conducted.

Patients who meet the selection criteria were subjected to the following:

1. Identification of cardiovascular risk factors (age, sex, smoking, hypertension, diabetes mellitus, dyslipidemia, body mass index, and family history of CAD).
2. Assessment of symptoms suggestive of coronary insufficiency as typical exertional chest pain and history of admission to coronary care unit with acute coronary syndrome whether unstable angina or MI.
3. Full clinical examination.
4. Resting 12 lead surface electrocardiogram to identify cardiac rhythm and signs of myocardial ischemia (ST segment and T wave abnormalities or pathological Q waves).
5. Echocardiography study was done using a 2.5-MHz transducer and harmonic imaging to measure systolic and end diastolic left ventricular dimensions, segmental wall motion abnormalities at rest, and left ventricle ejection fraction (LVEF) using biplane Simpson’s rule.
6. Treadmill stress electrocardiogram testing using Modified Bruce Protocol when indicated.
7. Laboratory testing includes cardiac biomarkers (CK-MB and troponin) measured at baseline and 8–12 h after the procedure and if indicated during the 12 months follow-up, fasting blood sugar, hemoglobin A1c level, lipogram, HCV antibodies using the third-generation ELISA method [33], liver function, and renal function tests.
8. Coronary angiography was performed by an experienced intervention cardiologist, using Seldinger’s technique where a right femoral artery puncture was done then right and left coronary angiogram was performed in multiple projections; coronary stenosis of more than 70% by visual assessment and documented ischemia was considered for inclusion [34]. The dual antiplatelet regimen consisted of aspirin in loading doses of 300 mg and maintenance of 100 mg/day maintained indefinitely and clopidogrel loading dose of 300–600 mg, followed by 75 mg/day for at least 1 year. After insertion of the arterial sheath to the right femoral artery, patients were administered 10,000 IU or 100–200 IU heparin/kg. PCI was performed using drug-eluting stents by an experienced interventionist who was blinded to clinical and demographic data. After 24-h hospital follow-up, the patient was discharged (if no complication occurred) with prescription of aspirin, clopidogrel, B-blockers, angiotensin-converting enzyme inhibitors or angiotensin receptor blockers, statin therapy, and other medications if indicated as anti-glycemics. Some patients received modified drug regimens due to associated different co-morbid conditions.

The primary end point was MACEs that include TLR, TVR, CABG, MI, CVS, stent thrombosis, major bleeding, and cardiac and non-cardiac death immediately and over a period of 12 months. The secondary end point was clinical in-stent restenosis indicated by the recurrence of ischemic chest pain, positive stress electrocardiogram, and new motion wall abnormality by echocardiography. Clinical in-stent restenosis was documented by coronary angiography if not contraindicated. The patients were followed up by clinic visits, at 3 months, 6 months, and 12 months to detect either the primary or secondary end points. Also, telephone communication was available for the patients at any time during the 12 months follow-up. Clinical in-stent restenosis is assessed as a requirement for ischemia-driven repeat revascularization; it was proposed by the Academic Research Consortium, this definition requires both an assessment of luminal narrowing and the patient’s clinical context.

The following formula was used to calculate the minimum size of the required sample:

$$ n={(z)}^2\ p\ \left(1-p\right)/{d}^2 $$

where *n* indicates the sample size, *z* indicates the level of confidence according to the standard normal distribution (for a level of confidence of 95%, *z* = 1.96), *p* indicates the estimated proportion of the population that presents the characteristic (about 10%), and *d* indicates the tolerated margin of error (for example, we want to know the real proportion within 5%).

Using the previous formula for the sample size calculation (*n*) = (1.96)^2^ × 0.10 (1 − 0.10)/(0.05)^2^ = 138.3. So, the minimum sample size is 139 participants.

### Participant characteristics

Patients aged ≥ 18 years who have symptomatic CAD and showed significant de novo stenotic lesions (≥ 70%) of native coronary arteries that were amenable to elective PCI using drug-eluting stents and who agreed and consented to participate in the study were included. We excluded patients who had any of the following criteria: target lesion in the left main coronary artery, patients with chronic total occlusion lesion, patients treated by primary PCI, patients who need bifurcation stenting technique, patients with previous history of CABG, known hypersensitivity to aspirin and clopidogrel, decompensated liver and kidney disease, congenital heart disease, chronic infections other than HCV infection, HCV-infected patients who received antiviral treatment, hematological or oncological disorders, and pregnancy.

### Analysis of data

Data was analyzed using SPSS version 20. Quantitative data were analyzed using the Student *t* test to compare means of two groups. Qualitative data were compared using Chi square test. Fisher’s exact correction was used when the expected cell count is less than 5. Univariate and multivariate Cox hazard regression analyses for MACEs and clinical in-stent restenosis at 12 months after adjustment for confounding factors were performed. *p* value was considered significant at or below 0.05.

The outcome variables were MACEs (TLR, TVR, CABG, MI, CVS, stent thrombosis, major bleeding, cardiac and non-cardiac death) as a primary end point and clinical in-stent restenosis as a secondary end point.

## Results

The study was conducted on 400 patients ≥ 18 years old who had CAD and are candidate for elective PCI using drug-eluting stents; they were classified into two equal groups: group 1 included HCV seropositive patients who did not received antiviral treatment, and group 2 included HCV seronegative patients.

### Baseline socio-demographic and clinical characteristics

There was a significantly higher number of males in group 1 than group 2 (146 vs 126, *p* value = 0.032); also, group 1 had a higher number of smokers than group 2 but was statistically insignificant (115 vs 101, *p* value = 0.16). There was no significant difference in the mean age of patients in both groups (51.21 ± 10.72 years versus 52.17 ± 10.39 years, *p* value = 0.521). Patients in group 2 had a significantly higher mean body mass index than that in group 1 (27.64 ± 3.81 vs 26.19 ± 2.9, *p* value = 0.003). As regards hypertension, diabetes mellitus, dyslipidemia, and family history of coronary artery disease in both groups, there was no statistically significant difference. As regards the clinical presentation, there was no statistically significant difference between the two groups (Tables 1 and 2).

**Table 1:** Baseline demographics and clinical characteristics

Variables	Group (1) (HCV seropositive) ***N*** = 200	Group (2) (HCV seronegative) ***N*** = 200	***P*** value
**Age**			
Mean ± SD (year)	51.21±10.72	52.17± 10.39	0.521
**Sex**			
Male	146 (73%)	126 (63%)	0.032
Female	54 (27%)	74 (37%)	
**Hypertension**			
Yes	118 (59%)	104 (52%)	0.159
**Diabetes mellitus**			
Yes	108 (54%)	108 (54%)	1.00
**Dyslipidemia**			
Yes	103 (51.5%)	115 (57.5%)	0.228
**Body mass index, Kg/m**^**2**^			
Mean± SD	26.19±2.9	27.64±3.81	0.003
**Smoking**			
Yes	115 (57.5%)	101 (50.5%)	0.16
**Family History of CAD**			
Positive	45 (22.5%)	40 (20%)	0.541

*Abbreviations*: *CAD* coronary artery disease, *HCV* hepatitis C virus, *N* number, *SD* standard deviation

**Table 2:** Baseline clinical presentations

Variables	Group (1)	Group (2)	***P*** value
**Stable Angina**			
Yes	34 (17%)	40 (20%)	0.44
**Unstable angina**			
Yes	112 (56%)	110 (55%)	0.841
**NSTEMI**			
Yes	54 (27%)	50 (25%)	0.648

*Abbreviations*: *NSTEMI* non-ST elevation myocardial infarction

### Baseline laboratory parameters

The baseline mean of serum low-density lipoprotein (LDL), hemoglobin A1c level, and LVEF in patients in both groups showed no statistically significant difference (Table 3).

**Table 3:** Baseline laboratory parameters

Variables	Group (1)	Group (2)	***P*** value
**Serum LDL**			
Mean± SD (mg/dl)	135.14±21.08	135.89±20.97	0.461
**Hemoglobin A1c**			
Mean± SD (%)	7.84±1.48	7.68±1.29	0.256
**LVEF**			
Mean± SD (%)	50.70 ± 9.57	49.95 ± 9.30	0.575

*Abbreviations*: *LDL* low density lipoprotein, *LVEF* left ventricle ejection fraction, *SD* standard deviation

### Baseline lesion and procedural characteristics

Patients in group 2 had a statistically significant higher grade TIMI flow on angiography than patients in group 1. Also, the mean length of stents implanted in group 2 was statistically significantly higher than in group 1, while the number of diseased vessels, mean percentage of luminal narrowing, number of stents implanted, mean stent diameter, and mean dilation pressure showed no statistically significant difference between both groups (Table 4).

**Table 4:** Baseline lesion and procedural characteristics

Variables	Group (1)	Group (2)	***P*** value
**N. of Diseased Vessels**			
Single vessel	106 (53%)	104 (52%)	0.678
Two vessels	53 (26.5%)	60 (30%)	
Three vessels	41 (20.5%)	36 (18%)	
**Percentage of luminal narrowing**			
Mean± SD (%)	84.80 ± 7.47	83.96 ± 7.30	0.259
**TIMI flow**			
Grade 2	33 (16.5%)	16 (8%)	0.01
Grade 3	167 (83.5%)	184 (92%)	
**N. of implanted stents**			
One stent	84 (42%)	88 (44%)	0.9
Two stents	70 (35%)	66 (33%)	
Three stents	46 (23%)	46 (23%)	
**Stent Length**			
Mean± SD **(mm)**	23.28 ± 6.72	25.24 ± 8.98	0.014
**Stent Diameter**			
Mean ± SD **(mm)**	3.11 ± 0.46	3.15 ± 0.40	0.326
**Dilation Pressure**			
Mean± SD **(atm)**	13.76 ± 3.98	13.48 ± 3.84	0.467

*Abbreviations*: *N* number, *SD* standard deviation

### Clinical outcomes of 24-h hospital stay follow-up

The incidence of MACEs was not statistically significantly different between both groups (3% vs 2.5%, *p* = 0.76). Also, the individual cardiovascular outcomes (emergency CABG, major bleeding, TLR, TVR, acute stent thrombosis, MI, CVS, cardiac and non-cardiac death) showed no statistically significant difference in both groups (Table 5).

**Table 5:** Clinical outcomes of 24-hour hospital stay follows up

Variables	Group (1)	Group (2)	***P*** value
**TLR**			
Yes	2 (1%)	1 (0.5%)	1.00
**TVR**			
Yes	0	0	
**Emergency CABG**			
Yes	0	0	
**CVS**			
Yes	0	1 (0.5%)	1.00
**MI**			
Yes	2 (1%)	1 (0.5%)	1.00
**Major bleeding**			
Yes	0	0	
**Cardiac death**			
Yes	2 (1%)	2 (1%)	1.00
**Non-Cardiac death**			
Yes	0	0	
**MACEs**			
Yes	6 (3%)	5 (2.5%)	0.76
**Acute Stent thrombosis**			
Yes	1 (0.5%)	1 (0.5%)	1.00

*Abbreviations*: *CABG* coronary artery bypass graft, *CVS* cerebrovascular stroke, *MACEs* major adverse cardiovascular events, *MI* myocardial infarction, *TLR* target lesion revascularization, *TVR* target vessel revascularization

### Clinical outcomes at 12 months follow-up

The cumulative incidence of MACEs (the primary end point) in patients of group 1 was slightly higher than that in group 2 (13.5% vs 11%, *p* value = 0.446) but statistically insignificant. Also, the individual cardiovascular outcomes (CABG, major bleeding, TLR, TVR, stent thrombosis, MI, CVS, cardiac and non-cardiac death) showed no statistically significant difference in both groups. Multivariate Cox hazard regression analyses for MACEs after adjustment of confounding factors (diabetes, hypertension, age, dyslipidemia, smoking, and number of implanted stents) revealed that HCV seropositivity did not present a higher hazard upon MACEs at 12 months follow-up (adjusted HR was 0.74; 95% CI 0.41–1.32; *p* value 0.302) (Tables 6 and 7).

**Table 6:** Cumulative incidence of MACEs at 12 months follow up period

Variables	Group (1)	Group (2)	***P*** value
**TLR**			
Yes	13 (6.5%)	11 (5.5%)	0.674
**TVR**			
Yes	3 (1.5%)	3 (1.5%)	1.00
**CABG**			
Yes	1 (0.5%)	2 (1%)	1.00
**CVS**			
Yes	2 (1%)	1 (0.5%)	1.00
**MI**			
Yes	5 (2.5%)	3 (1.5%)	0.72
**Major bleeding**			
Yes	0	0	
**Cardiac death**			
Yes	3 (1.5%)	2 (1%)	0.68
**Non-Cardiac death**			
Yes	0	0	
**MACEs**			
Yes	27 (13.5%)	22 (11%)	0.446
**Stent thrombosis**			
Yes	2 (1%)	2 (1%)	1.00

*Abbreviations*: *CABG* coronary artery bypass graft, *CVS* cerebrovascular stroke, *MACEs* major adverse cardiovascular events, *MI* myocardial infarction, *TLR* target lesion revascularization, *TVR* target vessel revascularization

**Table 7:** Multivariate Cox hazard regression analyses for MACEs at 12 months follow up

Variable	Unadjusted HR ratio (95% CI) for HCV seropositive patients versus seronegative (reference)			Adjusted HR ratio (95% CI) for HCV seropositive patients versus seronegative (reference)		
	HR	95% CI	*p* value	HR	95% CI	*p* value
**MACEs**	0.85	0.49-1.50	0.578	0.74	0.41-1.32	0.302

*Abbreviations*: *CI* confidence interval, *HCV* hepatitis C virus, *HR* hazard ratio, *MACEs* major adverse cardiovascular events

The cumulative incidence of clinical in-stent restenosis (all were documented by coronary angiography) (the secondary end point) was not statistically significantly different between both groups at 12 months (5.5% vs 5%, *p* value = 0.823). Multivariate Cox hazard regression analyses for clinical in-stent restenosis after adjustment of confounding factors (diabetes, age, dyslipidemia, smoking, and family history of CAD) revealed that HCV seropositivity did not present a higher hazard upon clinical in-stent restenosis at 12 months follow-up (adjusted HR was 1.70; 95% CI 0.64–4.51; *p* value 0.28) (Tables 8 and 9).

**Table 8:** Cumulative incidence of clinical in-stent restenosis at 12 months follow up period

Variables	Group (1)	Group (2)	***P*** value
**Clinical in-stent restenosis** (documented by coronary angiography)			
Yes	11 (5.5%)	10 (5%)	0.823

**Table 9:** Multivariate Cox hazard regression analyses for clinical in-stent restenosis at 12 months follow up

Variable	Unadjusted HR ratio (95% CI) for HCV seropositive patients versus seronegative (reference)			Adjusted HR ratio (95% CI) for HCV seropositive patients versus seronegative (reference)		
	HR	95% CI	*p* value	HR	95% CI	*p* value
**Clinical in-stent restenosis** (documented by coronary angiography)	0.96	0.41-2.27	0.93	1.70	0.64-4.51	0.28

*Abbreviations*: *CI* confidence interval, *HCV* hepatitis C virus, *HR* hazard ratio

## Discussion

It is postulated that infections adversely affect the cardiovascular system and so, it may adversely affect the PCI outcomes. To our knowledge, no previous study was carried out to determine the impact of HCV infection on the outcomes following PCI. Egypt has a high prevalence of HCV seropositive patients that may have an adverse impact on the PCI outcomes. Therefore, we conducted this study aimed to define the effect of HCV seropositivity on PCI outcomes, and we found that HCV seropositivity did not present a higher hazard upon MACEs compared to seronegative patients during the 24-h hospital stay and throughout the 12 months follow-up period. Also, HCV infection did not present a higher hazard upon clinical in-stent restenosis throughout the 12 months follow-up period compared to seronegative patients.

This study was in line with many previous studies that showed no association between HCV infection and cardiovascular diseases as Völzke et al. who conducted a cross-sectional study of the adult population in the northeast of Germany included 233 HCV Ab-positive patients and 4033 control individuals and showed no independent association between anti-HBs and anti-HCV antibody seropositivity and atherosclerotic endpoints such as prevalent myocardial infarction, stroke, carotid intima-media thickness, carotid plaques and stenos [35]. Also, Momiyama et al. reported in their study that there is a lack of any association between persistent hepatitis B or C virus infection and coronary artery disease [28]. Another study conducted by Arcari et al. investigated the association between HCV seropositivity and acute myocardial infarction in a case-control sample of men aged 30–50 years who were on active duty in the US Army during the period of 1991–2000 and found no relationship between HCV infection and coronary heart disease, but active duty military personnel tend to be in overall good health and have much lower age than the average age of a patient with acute myocardial infarction. Also, some of the control subjects in their study may have had an asymptomatic CAD [2]. The present study agrees with a study conducted by Kiechl et al. who reported that there is no association between chronic active hepatitis B virus and/or HCV infection and carotid plaque [29]. Also, Forde et al. failed to detect increased ACS risk in HCV-infected patients in their cohort study, but they did not exclude patients who had received antiviral treatment [30], which has been demonstrated to be related to lower cardiovascular hazard [36]. But in our study, we excluded patients on antiviral treatment, and all the participants already had established CAD, and we investigated the effect of HCV seropositivity on the outcomes following PCI which is a different situation to some extent.

On the other hand, many previous studies reported positive association between HCV seropositivity and CAD. Ishizaka et al. showed that there is an independent association between HCV seropositivity and the presence of carotid artery plaque [3]. Another study conducted by Ishizaka et al. assessed the role of HCV core protein and carotid atherosclerosis and reported a positive association between HCV core protein and carotid plaque, but only 1.3% of the participants tested positive for HCV core protein [37]. Also, Vassalle et al. showed that HCV seropositivity was an independent risk factor for coronary artery disease after adjusting for other confounding risk factors [16]. But the association showed in the previous 3 studies [3, 16, 37] between HCV infection and coronary artery disease was more significant than the associations shown for the well-established risk factors, including male sex, older age, hypertension, dyslipidemia, and smoking. It appears to be impossible that a relationship between HCV infection and CAD would be more grounded than a relationship with well-established risk factors. Butt et al. conducted a large national observational cohort study and reported that HCV-infected persons were younger and had lower lipid levels and a lower prevalence of hypertension, and despite a favorable risk profile, HCV infection was associated with a higher risk of CAD after adjustment for traditional risk factors, but they did not use adjudicated clinical measures for the diagnosis of CAD and did not consider some important risk factors for CAD as BMI and family history of CAD [15]. Another cohort study conducted by Tsai et al. reported that HCV-infected patients have a higher risk of developing ACS than non-infected patients do [17].

In contrary, Pothineni et al. reported that HCV-infected patients had less obstructive CAD on coronary angiography, but they studied a small number of patients (61 HCV-infected patients and 61 non-infected), and the patients were highly selected on the basis of antigen positivity. They did not consider the degree of HCV-induced liver disease and family history of CAD [32].

### Strengths of our study

First, to our knowledge, no previous study was carried out to determine the impact of HCV infection on the outcomes following PCI. The second, it was a multicenter study that included a large number of patients.

### Limitations of our study

First, we did not consider some important risk factors that may affect the outcomes as alcohol consumption, physical activity level, socioeconomic status, the patient’s lifestyle, complexity and severity of coronary artery lesions, site of coronary lesions, prescribed drug regimens for each patient, and drug adherence; all of which are potential confounding factors. Second, we included all HCV seropositive participants, regardless of the viral load which may play a role. Finally, degree of HCV-induced liver disease was not considered.

## Conclusion

HCV seropositivity had no effect on the cumulative incidence of MACEs, individual cardiovascular outcomes (TLR, TVR, MI, CVS, stent thrombosis, major bleeding, CABG, cardiac or non-cardiac death), or in-stent restenosis following PCI at 1 year follow-up period compared to seronegative patients. Therefore, it might be concluded that there was no impact of HCV seropositivity on the cardiovascular outcomes or clinical in-stent restenosis following PCI.

## Data Availability

The datasets used and/or analyzed during the current study are available from the corresponding author on reasonable request.

## Abbreviations

CABG: Coronary artery bypass graft
CAD: Coronary artery disease
CI: Confidence interval
CVS: Cerebrovascular stroke
HCV: Hepatitis C virus
HR: Hazard ratio
LDL: Low-density lipoprotein
MACEs: Major adverse cardiovascular events
MI: Myocardial infarction
PCI: Percutaneous coronary intervention
SD: Standard deviation
TLR: Target lesion revascularization
TVR: Target vessel revascularization
